# Response to comment on "PuraStat for gastrointestinal bleeding management: Effective approach for endoscopic prevention and rescue therapy"

**DOI:** 10.1055/a-2550-6677

**Published:** 2025-04-08

**Authors:** Roberta Maselli, Leonardo Da Rio, Cesare Hassan, Alessandro Repici

**Affiliations:** 1 9268 Endoscopy Unit, Department of Gastroenterology, IRCCS Humanitas Research Hospital, Rozzano, Milan, Italy; 2 437807 Department of Biomedical Sciences, Humanitas University, Pieve Emanuele, Milan, Italy



We thank Dr. Merola and colleagues for the thoughtful commentary about our study regarding use of PuraStat in endoscopic management and prevention of gastrointestinal bleeding. We appreciate the valuable insights shared, particularly the agreement with the potential of PuraStat for both managing active “oozing” bleeding and preventing delayed rebleeding.

Regarding the five cases of severe active bleeding discussed in our manuscript, we can provide some additional details to clarify management of these cases. Specifically:

- Two cases of active bleeding from duodenal ulcers were treated in the first instance with epinephrine injection and hemoclip placement.
- One case of active bleeding following a surgical colo-colonic anastomosis was treated in the first instance with hemoclip placement.
- One case of active bleeding following a left colonic endoscopic mucosal resection was treated in the first instance with hemoclip placement.
- One case of active bleeding that occurred during necrosectomy of a walled-off pancreatic necrosis was treated in the first instance with mechanical hemostasis.

As indicated in the manuscript, PuraStat was used as a secondary treatment in these cases, alongside other hemostatic techniques, primarily to prevent delayed rebleeding and ensure technical success. We recognize the importance of further clarifying the characteristics of the active gastrointestinal bleeding lesions most likely to benefit from PuraStat and agree that additional data would be valuable. Furthermore, we concur with the observation that prospective comparative studies will be essential for establishing the definitive role of PuraStat in bleeding control, as well as its impact on clinical outcomes such as hospitalization length, morbidity, and mortality rates.

**Publication note**

Letters to the editor do not necessarily represent the opinion of the editor or publisher. The editor and publisher reserve the right to not publish letters to the editor, or to publish them abbreviated or in extracts.

